# Craniofacial Osteosarcoma—Pilot Study on the Expression of Osteobiologic Characteristics and Hypothesis on Metastasis

**DOI:** 10.3389/fonc.2020.00745

**Published:** 2020-06-23

**Authors:** Manuel Weber, Stephan Söder, Janina Sander, Jutta Ries, Carol Geppert, Marco Kesting, Falk Wehrhan

**Affiliations:** ^1^Department of Oral and Maxillofacial Surgery, Friedrich-Alexander University Erlangen-Nürnberg (FAU), Erlangen, Germany; ^2^Institute of Pathology, Fürth, Germany; ^3^Institute of Pathology, Friedrich-Alexander University Erlangen-Nürnberg (FAU), Erlangen, Germany

**Keywords:** craniofacial osteosarcoma, osteosarcoma of the jaw, hedgehog, macrophage polarization, Gli1, M1, M2

## Abstract

**Background:** Craniofacial osteosarcomas (COS) and extracranial osteosarcomas (EOS) show distinct clinical differences. COS show a remarkably lower incidence of metastases and a better survival. However, in contrast to EOS, they show a poor response to neoadjuvant chemotherapy. Tumor-associated macrophages and their polarization as well as developmental biological signaling pathways are possible candidates for explaining the clinical differences between COS and EOS. The aim of the study was to analyze differential expression of macrophage markers and important regulators of these pathways.

**Methods:** Twenty osteosarcoma cases (10 COS and 10 EOS) were immunohistochemically stained to assess CD68, CD11c, CD163, MRC1, Gli1, and Gli2 expression. Statistical differences between COS and EOS were tested using the Mann–Whitney *U* test. Additionally, the paper describes an example of multidisciplinary treatment of a patient suffering from COS and discusses the surgical challenges in treatment and rehabilitation of COS.

**Results:** COS showed a significantly (*p* < 0.05) increased infiltration of CD11c-positive M1 macrophages and a shift toward M1 polarization compared to EOS. Additionally, COS revealed a significantly (*p* < 0.05) lower Gli1 expression than EOS.

**Conclusion:** The reduced Gli1 expression in COS can be interpreted as reduced activation of the Hedgehog (Hh) signaling pathway. The increased M1 polarization and reduced Hh activation in COS could explain the low incidence of metastases in these osteosarcomas.

## Introduction

Osteosarcomas are the most frequent primary bone tumors (1). Osteosarcomas are affecting predominantly young people and are characterized by a poor prognosis and yet unsatisfying therapeutic options. The early formation of metastases is the outstanding clinical problem and, in many cases, the limiting factor for the patient (2, 3).

Craniofacial osteosarcomas (COS) represent an exception in this regard. Although, due to local progression, they are also characterized by an unfavorable prognosis, formation of metastases is an extremely rare event in these tumors (1, 4–6). Besides the different metastatic behavior, there are several other clinical differences between craniofacial (COS) and extracranial osteosarcomas (EOS). While the 5-year survival of COS is ~77%, EOS show a worse 5-year survival of only about 55–70% (1, 4). The introduction of neoadjuvant chemotherapy 30 years ago revolutionized the treatment of EOS. Before the introduction of chemotherapy, over 90% of patients with extracranial osteosarcoma died from distant metastases (7). With polychemotherapy, an increase in cure rates from only ~10 to 60–70% could be achieved (4). In contrast, the role of chemotherapy in craniofacial osteosarcomas is still unclear, and meta-analyses have reported conflicting results (3, 4). There are also data showing that treatment with surgery alone was associated with significantly longer survival rates than surgery with adjuvant chemotherapy in COS (1, 3, 8). With a typical occurrence in the third and fourth decade of life, COS patients are usually older than EOS cases (4). The most frequent COS are osteosarcomas of the jaw (3, 4).

**Figure 1:**
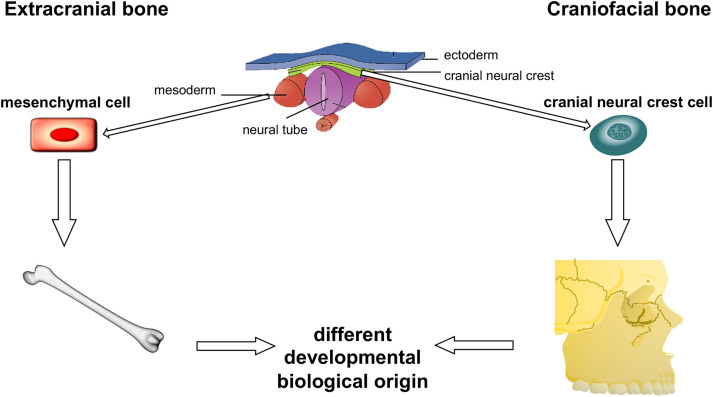
Developmental biological origin of craniofacial and extracranial bone. The figure shows the different developmental biological origin of the craniofacial and extracranial bones. Extracranial bone is derived from the mesenchyme, whereas the craniofacial bone originates the cranial neural crest. The cranial neural crest is of ectodermal origin. (The figure was created adopting the neurulation scheme from Anatomy & Physiology, Connections: Web site. http://cnx.org/content/col11496/1.6/, Jun 19, 2013 and using the software tool powerpathways, 2010; source: epath3d San Diego, epath3d.com).

Compared to extracranial bone, craniofacial bone shows several special characteristics: A faster turnover and remodeling and the relative absence of osteoporosis can be observed in craniofacial bone (9, 10). Furthermore, a different expression of osseous differentiation markers was reported by several studies (10–12). To understand the special features of the craniofacial bone, the special embryologic development has to be considered. In contrast to the axial skeleton, craniofacial bone does not derive from mesenchymal progenitor cells. Instead, craniofacial bone derives from the cranial neural crest, which represents neuroectodermal tissue (13, 14) (Figure 1).

This different embryologic origin of craniofacial and extracranial bones could explain clinically observed differences between COS and EOS. The Hedgehog (Hh) pathway plays a critical role in embryonic development and in pathogenesis of human tumors (15). Loss-of-function mutations in the Hedgehog receptor Patched (PCT) or gain-of-function mutations in the signal transduction protein Smoothened (SMO) activate Hh signaling. Smoothened inhibitors like Vismodegib are already used in the routine therapy of advanced basal cell carcinoma (16). Hh signaling finally leads to the activation of the transcription factors Gli1, Gli2, and Gli3, which are differentially expressed in different tissues.

A high Gli2 expression could be shown in osteosarcoma cell lines, and a correlation of Gli2 expression with the prognosis of osteosarcoma patients was reported (15). *In vitro*, Gli2 inhibition led to a reduced proliferation of tumor cells and an increased sensitivity to chemotherapeutic agents (15). In chondrosarcomas and Ewing sarcomas, the involvement of the Hh pathway in tumorigenesis is also shown (16). The role of the Hh signaling pathway in COS is not yet investigated. However, Hh signaling plays a critical role in craniofacial embryologic development. It is shown that patterning of the cranial neural crest and facial morphogenesis require Hh signaling (17).

Differences in tumor immunology are another possible explanation for the diverse clinical behavior of COS and EOS. In this regard, tumor-associated macrophages could be of particular relevance, as they account for up to 50% of the tumor volume in some malignancies (2). An explorative gene expression analysis showed that EOS cases with and those without metastasis within 5 years differ regarding the expression of genes associated with regulation of macrophage functions (18). Macrophages play a key role in the progression and metastasis of most solid tumors (19–22). In breast cancer, for example, macrophages are involved in the growth of bone metastases (2) and may influence chemotherapy response (23). The influence of macrophages on osteosarcomas has not yet been conclusively understood. There are studies showing an association between high macrophage infiltration and unfavorable prognosis (24). Other studies, however, come to the opposite conclusion (18). Studies regarding tumor-associated macrophages in COS are lacking so far.

Currently, there are no data available in the literature, describing the different tumor biological behavior of osteosarcomas depending on their primary location (craniofacial vs. extracranial).

The exception of craniofacial osteosarcomas could help identifying the molecular factors facilitating the metastases of osteosarcomas and may lead to new therapeutic interventions. The current pilot study aims to test if COS and EOS differ regarding macrophage infiltration, macrophage polarization, and activation of Hedgehog signaling.

## Materials and Methods

### Patients and Tissue Harvesting

For this retrospective analysis, tissue specimens of 10 cases of craniofacial osteosarcomas (COS) and 10 cases of extracranial osteosarcomas (EOS) treated at the university hospital of Erlangen during 2005 and 2015. The study was approved by the ethics committee of the Friedrich-Alexander University Erlangen–Nürnberg (70_15 Bc) and performed in accordance with the Declaration of Helsinki. There was an equal distribution between male and female patients. The mean age was 40.6 years in the COS group and 26.5 years in the EOS group. Metastatic disease was present at the time of surgery or in the follow-up in one COS case and in eight EOS cases. Most osteosarcomas were high-grade sarcomas. Five COS cases were osteosarcomas of the mandible and five cases osteosarcomas of the maxilla. The demographic characteristics are given in Table 1.

**Table 1:** Demographic parameters of the patient cohort.

	**Description of the patient collective; total number of cases: 20**				
		**COS**		**EOS**	
		***n***	**% of cases**	***n***	**% of cases**
Number of cases		10		10	
Gender	Male	5	50%	4	40%
	Female	5	50%	6	60%
Mean age		40.6 years (SD 18.2)		26.5 years (SD 19.2)	
Age range		19–75 years		5–63 years	
Analyzed specimen	Primary tumor	8	80%	10	100%
	Recurrence	2	20%	0	
Metastatic disease	Yes	1	10%	8	80%
	No	9	90%	2	20%
Grading	G1	1	10%	0	0%
	G2	2	20%	1	10%
	G3	4	40%	7	70%
	Unknown	3	30%	2	20%

*Gender, age at diagnosis, grading, and presence of metastatic disease are displayed*.

*COS, craniofacial osteosarcomas; EOS, extracranial osteosarcomas*.

### Immunohistochemical Staining and Quantitative Analysis

Established antibodies were used to detect macrophage infiltration and macrophage polarization. CD68 is an established pan-macrophage marker to detect macrophages independent of their polarization (25–27). M1-polarized macrophages express the CD11c antigen (27–29). M2-polarized macrophages express the CD163 (25, 26, 30, 31) and the MRC1 antigen (28, 30, 32). The immunohistochemical staining procedure was performed as previously described (21, 33). Gli1 and Gli2 staining was performed after samples were treated for 20 min with the detergent TritonX (Merck, Darmstadt, Germany) to enable better nuclear penetration of the antibodies. The following primary antibodies were used: anti-CD68 (11081401, clone KP1, Dako, Hamburg, Germany), anti-CD11c (ab52632, clone EP1347y, Abcam, Cambridge, UK) anti-CD163 (NCL-CD163, 6027910, Novocastra, Newcastle, USA), anti-MRC1 (H00004360-1102, clone 5C11, Abnova), anti-Gli1 (ab151796, 1:200, Abcam, Cambridge, UK), and anti-Gli2 (ab7181, 1:200, Abcam, Cambridge, UK).

An appropriate positive control was included in each series.

The tumor and biopsy sections were completely scanned and digitized using the method of “whole slide imaging.” The scanning procedure was performed in cooperation with the Institute of Pathology of the University of Erlangen–Nürnberg using a Pannoramic 250 Flash III Scanner (3D Histech, Budapest, Hungary) and in 40× magnification mode. All samples were digitally analyzed (Case viewer, 3D Histech, Budapest, Hungary). Quality controls were performed under a bright-field microscope (Zeiss Axioskop and Axiocam 5, at 10–40× magnification). H&E-stained sections of all samples were examined together with a pathologist to ensure that all samples contained representative osteosarcoma tissue.

For each sample and each marker, three visual fields showing the highest infiltration rate of positive cells were selected (hot spot analysis). The complete area of all three visual fields of one specimen was between 1.1 and 1.5 mm^2^ (Case viewer, 3D Histech, Budapest, Hungary).

Micrographs of the selected areas were imported into the BioMas analysis software (modular systems of applied biology, Erlangen, Germany) for cell counting.

A quantitative analysis was performed to determine the numbers of CD68-, CD11c-, CD163-, MRC1-, Gli1- and Gli2-positive cells in the osteosarcoma tissue. Assessment of the cell density per square millimeter was performed as previously described (22, 33).

### Statistical Analysis

To analyze the immunohistochemical staining, the cell count per square millimeter was determined as the number of positive cells per square millimeter of the specimen. Labeling index was calculated by dividing the number of positive cells by the number of all cells (positive + negative). The results are expressed as the median and standard deviation (SD). Box plot diagrams represent the median, the interquartile range, minimum (Min), and maximum (Max).

Two-sided, adjusted *p* ≤ 0.05 were considered to be significant. The analyses were performed using the Mann–Whitney *U* test with SPSS 22 for Mac OS (IBM Inc., New York, USA).

## Results

### Macrophage Infiltration and Polarization in COS and EOS

**Figure 2:**
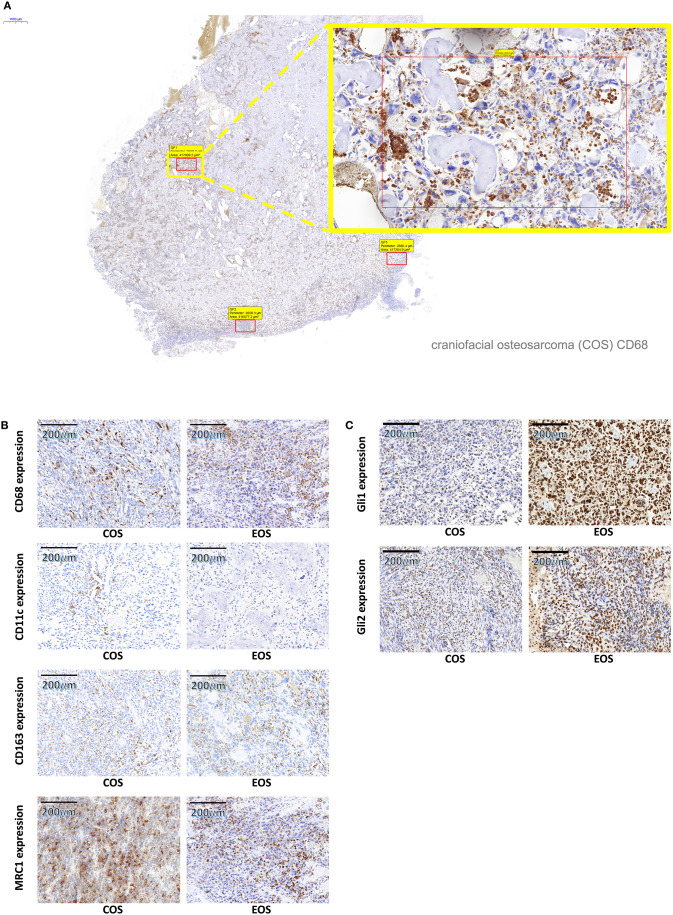
Typical macrophage marker and Gli staining pattern. **(A)** shows exemplarily the typical expression pattern of the generic macrophage marker CD68 in a craniofacial osteosarcoma. CD68-positive cells are stained in brown. A panoramic view (2× magnification) is given on the left side, and a magnification of the indicated region (25× magnification) is displayed on the right side. Three fields of view are marked in the panoramic micrograph for cell counting. **(B)** shows high power micrographs (35× magnification) of CD68, CD11c, CD163, and MRC1-positive macrophages in COS and EOS. All macrophage markers reveal acytoplasmic and membranous expression pattern. **(C)** shows high power micrographs (35× magnification) of Gli1- and Gli2-positive tumor cells in COS and EOS. Both markers reveal a nuclear expression pattern. COS, craniofacial osteosarcomas; EOS, extracranial osteosarcomas.

The analyzed macrophage markers CD68, CD11c, CD163, and MRC1 showed a staining of the plasma membrane and the cytoplasm, as it was already described (33). In addition to mononucleated cells, polynuclear osteoclasts also expressed macrophage markers. An example of the staining pattern of macrophage markers is given in Figures 2A,B.

**Figure 3:**
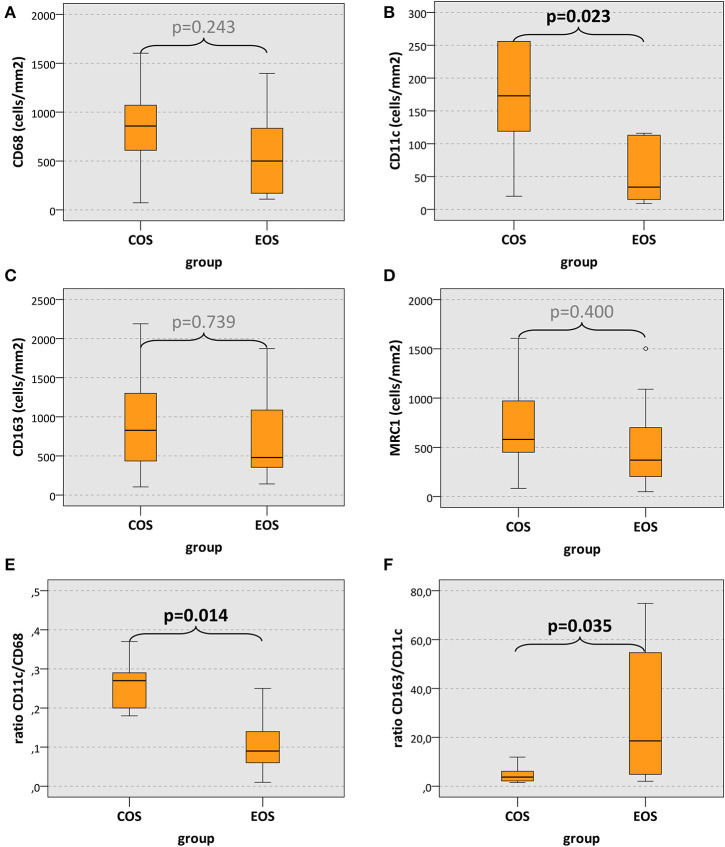
Macrophage cell count and macrophage expression ratios. **(A–D)** The box plots show macrophage infiltration (positive cells/mm^2^) and **(E,F)** macrophage expression ratios in craniofacial osteosarcomas (COS) and extracranial osteosarcomas (EOS). *p* values generated by the Mann–Whitney *U* test are given. Significant *p* values are printed in “bold” letters.

CD68 cell count in COS was increased compared to EOS without reaching statistical significance (median, 858 and 500 cells/mm^2^, respectively) (*p* = 0.243) (Table 2, Figure 3A). However, CD11c expression in COS cases was significantly higher than in EOS (median, 173 and 34 cells/mm^2^, respectively) (*p* = 0.022) (Table 2, Figure 3B). There was no significant difference in CD163 and MRC1 expression between COS and EOS (Table 2, Figures 3C,D).

**Table 2:** Macrophage cell count (positive cells/mm^2^) and the macrophage marker expression ratio in craniofacial (COS) and extracranial osteosarcomas (EOS).

	**Macrophage infiltration, macrophage expression ratios, and Gli expression in craniofacial osteosarcomas (COS) and extracranial osteosarcomas (EOS)**				
		***n***	**Median**	**SD**	***p* value**
**Macrophage infiltration**					
CD68	COS	10	858	449	0.243
(cells/mm^2^)	EOS	10	500	429	
CD11c	COS	10	173	211	0.022
(cells/mm^2^)	EOS	10	34	261	
CD163	COS	10	828	637	0.739
(cells/mm^2^)	EOS	10	480	609	
MRC1	COS	10	580	456	0.400
(cells/mm^2^)	EOS	10	370	480	
**Macrophage expression ratios**					
Ratio	COS	10	0.27	0.13	0.014
CD11c/CD68	EOS	10	0.09	0.48	
Ratio	COS	10	1.04	0.55	0.447
CD163/CD68	EOS	10	1.48	1.03	
Ratio	COS	10	3.75	3.53	0.035
CD163/CD11c	EOS	10	18.54	28.36	
Ratio	COS	10	3.43	1.88	0.182
MRC1/CD11c	EOS	10	6.04	26.19	
**Gli expression**					
Gli1	COS	10	1,102	676	0.035
(cells/mm^2^)	EOS	10	2,883	1,307	
Gli1	COS	10	0.24	0.21	0.028
Labeling index	EOS	10	0.72	0.23	
Gli2	COS	10	3,217	1,441	0.829
(cells/mm^2^)	EOS	10	3,319	1,510	
Gli2	COS	10	0.65	0.14	0.101
Labeling index	EOS	10	0.84	0.24	

*Additionally, the Gli1 and Gli2 expression (positive cells/mm^2^ and labeling index) in COS and EOS is given. Values represent the median, standard deviation (SD), and p value (Mann–Whitney U test). n, number of cases*.

The ratio between CD11c-expressing cells and CD68-positive cells (CD11c/CD68 ratio; indicator of M1 polarization) in COS cases was significantly higher (median value, 0.27) than in EOS cases (median value, 0.09) (*p* = 0.014) (Table 2, Figure 3E). Accordingly, the CD163/CD11c ratio (indicator of M2 polarization) in COS was significantly lower than in EOS (median value, 3.75 and 18.54, respectively) (*p* = 0.035) (Table 2, Figure 3F). The MRC1/CD11c ratio and the CD163/CD68 showed no statistically significant difference (Table 2).

### Gli Expression in COS and EOS

Gli1 and Gli2 showed expression predominantly in the nuclear compartment of osteosarcoma tumor cells (Figure 2C).

**Figure 4:**
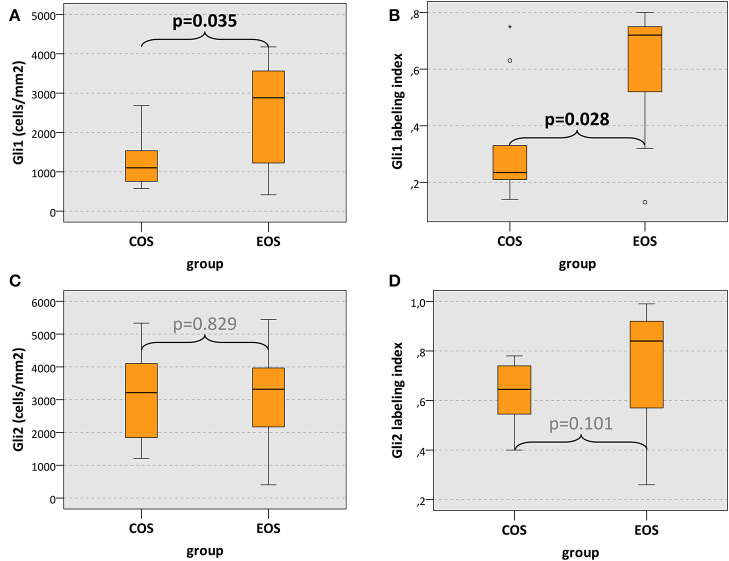
Gli1 and Gli2 expression. **(A,C)** The box plots show Gli1 and Gli2 expression displayed as cell density (positive cells/mm^2^) and **(B,D)** labeling index (percentage of expressing cells). Values for craniofacial osteosarcomas (COS) and extracranial osteosarcomas (EOS) are given. *p* values are generated by the Mann–Whitney *U* test. Significant *p* values are printed in “bold” letters.

Gli1 cell count (positive cells/mm^2^) in COS was significantly lower compared to EOS (median, 1,102 and 2,883 cells/mm^2^, respectively) (*p* = 0.035) (Table 2, Figure 4A). Additionally, the Gli1 labeling index (positive cells/all cells) in COS was significantly lower than in EOS (median value, 0.24 and 0.72, respectively) (*p* = 0.028) (Table 2, Figure 4B). In contrast, there was no significant difference in Gli2 expression between COS and EOS (Table 2, Figures 4C,D).

## Discussion

### Role of Macrophage Polarization in COS and EOS

COS cases showed an increased infiltration of macrophages. However, only the M1 macrophage marker CD11c (27–29) showed significantly increased cell density in COS cases. Macrophages can have two different activation sets or polarizations: M1 and M2 (34–36). M1 macrophages promote inflammatory reactions, are capable of antigen presentation and T-cell activation, and have therefore antitumor and antimetastatic effects (34–36). M2 macrophages have immunoregulatory properties and are associated with wound healing, immunosuppression, tumor progression, and metastatic spread (20, 21, 25, 28, 34–39).

In addition to the significantly increased CD11c cell density in COS, we could show a significantly increased CD11c/CD68 ratio in COS cases. The CD11c/CD68 ratio can be seen as indicator of M1 polarization (40). Accordingly, the CD163/CD11c ratio—as indicator of M2 polarization—was significantly higher in EOS. These results suggest that there might be an increased degree of M1 polarization of macrophages in COS compared to EOS. In EOS, an association of M1 polarization of macrophages and high macrophage infiltration with low incidence of metastases and better outcome was already shown (41). These data are in accordance with the results of the current study in which we could show an increased degree of M1 polarization and a tendency towards increased macrophage infiltration in COS.

It is shown that muramyl tripeptide phosphatidyl ethanolamine (MTP-PE) can be used for the adjuvant treatment of osteosarcoma (42, 43). MTP-PE acts by increasing M1 polarization of macrophages (43). While meta-analyses showed no clear benefit for adjuvant MTP-PE treatment for overall survival, there was a positive effect for cases with absence of metastases reported (44). This indicates a potential metastasis preventing effect through M1-polarized macrophages. A combination of MTP-PE with bisphosphonates was shown to be a potential candidate for adjuvant EOS treatment (42). This is interesting as bisphosphonates also have M1 polarizing properties (45). Additionally, a prevention of osteosarcoma metastases by antagonizing M2 polarization of macrophages with all-trans-retinoic acid was shown (46).

If the increased degree of M1 polarization in COS suggested by this pilot study can be verified in confirmatory analyses, it needs to be assessed if macrophage modulating treatments are exclusively beneficial for EOS cases or if COS with an inherent increase in M1 polarization can also profit from such immune modulatory approaches.

### Hedgehog Signaling in COS and EOS

The Hedgehog (Hh) pathway plays a relevant role in the progression and metastatic spread of several cancers including osteosarcomas (47). Hh target genes are involved in proliferation, survival, stem cell formation, and invasion (47). Increased Hh signaling in osteosarcomas was associated with inferior survival and metastatic disease (47, 48).

**Figure 5:**
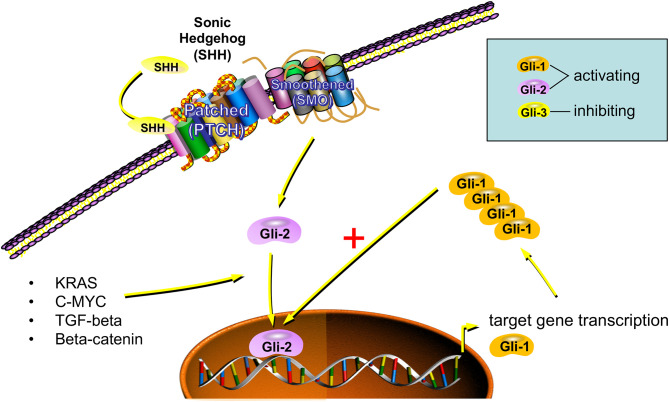
Interpretation of increased Gli1 expression as indicator of increased hedgehog signaling. Binding of a hedgehog ligand-like sonic hedgehog (SHH) to the transmembrane receptor Patched (PTCH) leads to the dissociation of PTCH from Smoothened (SMO). Thereby signal transduction to the cytoplasm is initiated, and Gli transcription factors are translocated to the nucleus. Gli2 is the main factor responsible for the transcription of hedgehog target genes. Gli1 is one of the hedgehog target genes and its transcription is increased by hedgehog signaling. Gli1 augments the transcription activation of Gli2. In this context, Gli1 expression can be interpreted as surrogate marker of hedgehog activation. Besides the described canonical hedgehog activation through extracellular ligand-like sonic hedgehog (SHH), a noncanonical hedgehog activation via oncogenic pathways like KRAS, C-MYC, transforming growth factor beta (TGF-beta), or beta-catenin can also be observed (The figure was created using the software tool powerpathways, 2010; source: epath3d San Diego, epath3d.com).

The endpoint of intracellular Hh signaling is the activation of Gli transcription factors. Gli1 and Gli2 act as transcriptional activators, while Gli3 is a transcriptional repressor (48). The current pilot study could show that COS have a significantly reduced Gli1 expression compared to EOS. However, there was no significant difference regarding Gli2 expression detected. In this regard, it needs to be noted that Gli1 is one of the target genes of the Hh pathway and therefore can act as indicator of Hh activation (48, 49). The increased Gli1 expression in cells with activated Hh signaling can then be detected by immunohistochemical staining. An overview of Hh signaling in osteosarcoma cells is given in Figure 5.

It was shown that an inhibition of Hh signaling inhibits proliferation, migration, and invasion of osteosarcoma cells *in vitro* (50). As a result of Hh inhibition a decreased cellular Gli1 expression was reported (50). An antimetastatic effect of Hh inhibition was verified in an animal model in which lung metastases and tumor growth were inhibited (50). A combination of standard chemotherapy with Hh inhibitors was shown to synergistically prevent osteosarcoma progression *in vivo* and could also be used for human treatment (51). In this regard, the rare occurrence of metastatic disease could be associated with the decreased degree of Hh activation in COS compared to EOS. These data indicate that Hh inhibition might be a promising therapeutic approach for EOS.

However, an increased radioresistance of osteosarcoma cells was reported to be associated with high Hh activation and could be reversed by Hh inhibition (52, 53). In this regard, Hh inhibition might also be considered for new studies evaluating multimodal treatment including radiotherapy in COS.

Besides the canonical Hedgehog activation via extracellular ligands like Sonic Hedgehog (SHH), there is also a noncanonical Hedgehog activation via oncogenic pathways like KRAS, C-MYC, transforming growth factor beta (TGF-beta), or beta-catenin described (Figure 5) (54). In this regard, it needs to be evaluated if Hh inhibition on the level of the transmembrane receptors is sufficient for osteosarcoma therapy. However, it could be shown that several Smoothened inhibitors are sufficient to inhibit Gli1 expression and proliferation in osteosarcoma cell lines (55).

The results of the current pilot study indicate that Hh activation in COS might be reduced compared to EOS. This could explain the low incidence of metastases in COS and supports the investigation of Hh inhibitors in osteosarcoma treatment.

### Limitations of the Study

The main limitation of the study is the low number of analyzed cases. In this regard, it needs to be considered that COS are relatively rare tumors. Most centers in Germany treat about one case a year. The current pilot study could motivate a larger multicenter analysis in the future.

A further limitation is the lack of specificity of the available macrophage marker. This aspect is already discussed elsewhere (33). The current study uses the Gli transcription factors as surrogate markers for the activation of the hedgehog signaling pathway. An analysis of hedgehog ligands, receptors, and further target genes would be desirable in future analyses.

## Conclusion

The current pilot study could show that Hedgehog activation in COS is significantly lower than in EOS. This finding could be caused by the different developmental biological origin of craniofacial and extracranial bone and could contribute to the low incidence of metastases in COS. The shift of macrophage polarization towards the antimetastatic M1 type could also contribute to the uncommon metastatic spread in COS.

Based on these tumor biological differences, the diverse metastatic behavior, and the clinical response to chemotherapy, COS and EOS should be considered as different tumor entities that also require a specific treatment regime. Thus, the therapeutic concept of EOS cannot simply be transferred to COS. Prospective studies are needed to evaluate the value of adjuvant therapy in COS treatment. For COS, surgical resection with wide margins is currently the only available treatment with a high level of evidence. As a result, functionally important anatomical structures of the orofacial tissue often have to be sacrificed. Therefore, the anatomic reconstruction is essential to preserve the quality of life of patients.

## Data Availability Statement

The datasets generated for this study are available on request to the corresponding author.

## Ethics Statement

The studies involving human participants were reviewed and approved by ethics committee of the Friedrich-Alexander University Erlangen-Nürnberg. Written informed consent for participation was not required for this study in accordance with the national legislation and the institutional requirements. Written informed consent was obtained from the individual(s) for the publication of any potentially identifiable images or data included in this article.

## Author Contributions

MW formulated the hypothesis, applied for grant support (VFWZ Germany), initiated and conducted the study, interpreted the data and wrote the manuscript. FW formulated the hypothesis, created the Figures 1, 5, interpreted the data and contributed relevantly to the manuscript. SS selected the patients, performed the histologic analysis of all samples, helped to validate the markers, contributed to the discussion and critically reviewed the manuscript. JS collected the tissue samples, performed the macrophage stainings, interpreted the data, and contributed to the manuscript. JR and MK contributed to the discussion and critically reviewed the manuscript. CG performed the digitalization of the specimens, helped with cell counting and critically reviewed the manuscript. All authors read and approved the final manuscript.

## Conflict of Interest

The authors declare that the research was conducted in the absence of any commercial or financial relationships that could be construed as a potential conflict of interest. The handling editor declared a shared affiliation, though no other collaboration, with the authors at the time of review.
